# Spectral-Distribution Uncertainty Modeling for Robust Cross-Domain Medical Image Segmentation

**DOI:** 10.3390/bioengineering13101163

**Published:** 2026-10-05

**Authors:** Chao Xin, Zhixiong Chen, Zeyu Huang, Jiucun Wang, Luojun Lin

**Affiliations:** 1 State Key Laboratory of Genetics and Development of Complex Phenotypes, School of Life Sciences and Human Phenome Institute, Fudan University, Shanghai 200433, China; 23110700156@m.fudan.edu.cn; 2 Medical Informatics Center, The First Affiliated Hospital of Ningbo University, Ningbo 315010, China; 3 College of Computer and Data Science, Fuzhou University, Fuzhou 350100, China; yuanczx@foxmail.com (Z.C.); zeyuhuang02@163.com (Z.H.)

**Keywords:** medical image segmentation, uncertainty modeling, spectral distribution

## Abstract

Domain generalization (DG) for medical image segmentation is commonly approached by simulating domain shifts through deterministic image transformations or feature perturbations. However, such methods implicitly assume that unseen domains can be approximated by predefined appearance variations. In this work, we challenge this assumption and hypothesize that medical domain shifts are more fundamentally characterized as uncertainty in spectral distributions. While anatomical structures are largely preserved across institutions, scanners, and acquisition protocols, substantial variations arise in image appearance, texture, and artifacts, which are predominantly manifested in the spectral domain. To test this hypothesis, we propose Spectral-Distribution Uncertainty (SDU), a frequency-aware DG framework that explicitly models uncertainty in the Fourier amplitude distributions of intermediate representations. By decomposing Transformer features into amplitude and phase components, SDU disentangles domain-specific spectral characteristics from domain-invariant anatomical semantics. Instead of imposing handcrafted frequency perturbations, SDU estimates spectral-distribution uncertainty during training and injects stochastic variations into the amplitude space, synthesizing diverse yet anatomically consistent representations that emulate potential unseen domains. Consequently, SDU expands the support of spectral feature distributions, enabling the model to learn representations that are robust to uncertainty-induced spectral shifts. Extensive experiments on retinal fundus and prostate MRI benchmarks demonstrate that SDU consistently outperforms state-of-the-art DG methods. Beyond empirical gains, these findings provide evidence that medical domain shifts are more appropriately characterized as uncertainty in spectral feature distributions, offering a principled perspective for generalizable medical image segmentation.

## 1. Introduction

Deep learning has achieved remarkable success in medical image segmentation and has become a vital component of numerous clinical applications [1,2,3]. Despite their impressive performance on in-domain data, segmentation models often suffer significant performance degradation when deployed across unseen clinical environments [4]. Such degradation is primarily attributed to domain shifts caused by imaging variations between training and deployment environments. However, it is impractical to collect and annotate data from every possible deployment environment. Therefore, domain generalization (DG), which aims to learn models that generalize to unseen domains without access to target-domain data during training, has emerged as an important research topic in medical image analysis [5].

Existing medical DG approaches primarily address domain shifts through image-level augmentation [6,7], style randomization [8,9], network optimization [10,11], or distribution alignment [12]. Although these methods have obtained promising performance, they share an implicit assumption: unseen domains can be approximated by deterministic transformations of observed source data. Consequently, existing methods attempt to simulate potential target domains through predefined appearance variations or handcrafted perturbation strategies. However, such an assumption may be overly restrictive for medical imaging scenarios. In practice, domain shifts stem from a complex combination of various factors, including scanner configurations, acquisition protocols, reconstruction pipelines, and imaging artifacts, making unseen domains difficult to characterize through a fixed set of transformations. This raises a fundamental question: are medical domain shifts truly deterministic appearance variations, or do they reflect a deeper form of distribution uncertainty?

In this work, we revisit medical domain shifts from a spectral perspective. We observe that while anatomical structures remain largely preserved across institutions, scanners, and imaging protocols, substantial variability emerges in image appearance, texture, noise patterns, and reconstruction artifacts. Importantly, these variations are predominantly reflected in spectral characteristics rather than anatomical content. This observation suggests that unseen domains may be better understood as stochastic realizations of latent spectral distributions instead of deterministic transformations of source-domain data. Based on this insight, we hypothesize that medical domain shifts are fundamentally characterized by uncertainty in spectral feature distributions. If this hypothesis holds, then improving domain generalization requires not merely generating more appearance variations, but explicitly modeling and learning invariance to spectral-distribution uncertainty.

**Figure 1:**
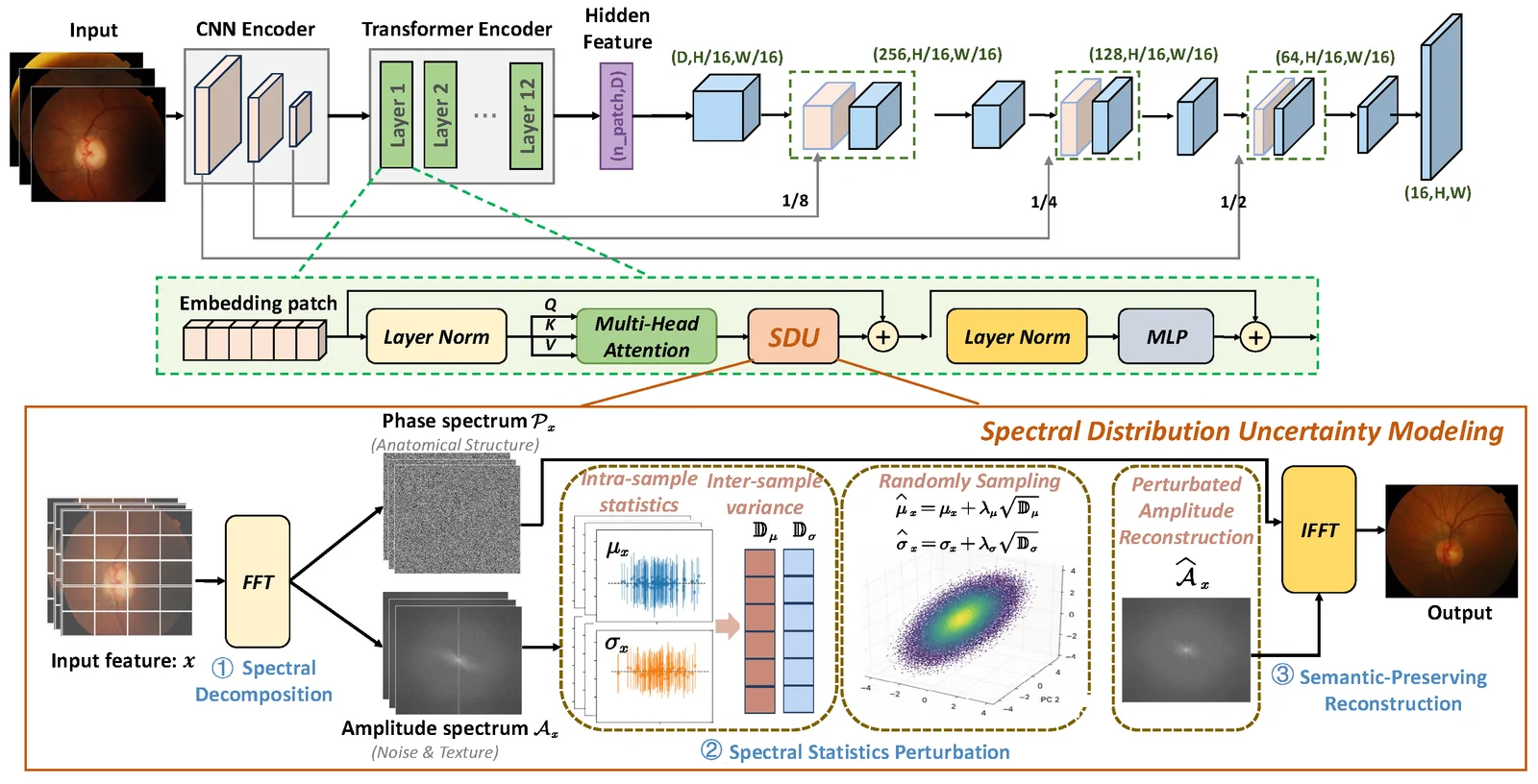
Proposed framework integrating TransUNet architecture with **SDU** module. (1) Spectral decomposition of intermediate Transformer encoder features (anatomical structure in phase spectrum vs. noise/texture in amplitude spectrum), (2) Spectral statistics perturbation via intra-sample statistics ($\mu_{x},\sigma_{x}$) and inter-sample variance ($D_{\mu },D_{\sigma }$), (3) Semantic-preserving reconstruction using perturbed amplitude and unchanged phase.

To investigate this hypothesis, we propose **Spectral-Distribution Uncertainty (SDU)**, a frequency-aware domain generalization framework for medical image segmentation. As shown in Figure 1, SDU decomposes intermediate Transformer representations into Fourier amplitude and phase components, enabling an explicit separation between domain-specific textural characteristics and domain-invariant anatomical semantics. Rather than relying on handcrafted frequency perturbations in prior studies [8], SDU estimates uncertainty in spectral amplitude distributions and injects stochastic variations accordingly, synthesizing diverse yet anatomically consistent feature representations that emulate potential unseen domains. Extensive experiments on retinal fundus and prostate MRI benchmarks demonstrate the effectiveness of the proposed framework. More importantly, the empirical findings provide evidence that modeling uncertainty in spectral feature distributions constitutes a principled mechanism for achieving robust generalization across heterogeneous clinical scenarios.

The main contributions are summarized as follows:

(1) We propose a new perspective on generalizable medical image segmentation by hypothesizing that domain shifts are fundamentally characterized by uncertainty in spectral feature distributions rather than deterministic appearance transformations.
(2) We introduce *Spectral-Distribution Uncertainty (SDU)*, a frequency-aware framework that explicitly models spectral-distribution uncertainty through stochastic amplitude-space perturbations.
(3) Extensive experiments across multiple medical segmentation benchmarks demonstrate superior generalization performance and provide empirical evidence supporting the proposed spectral uncertainty hypothesis.

## 2. Related Work

### 2.1. Domain Generalization in Medical Image Segmentation

Medical image segmentation models are often trained under the assumption that training and testing samples follow similar distributions. However, in real clinical scenarios, images collected from different scanners, institutions, acquisition protocols, and patient populations usually exhibit substantial appearance shifts. Domain generalization (DG) aims to improve model robustness on unseen target domains without accessing target-domain data during training, and has therefore attracted increasing attention in medical image segmentation. Existing studies can be roughly grouped into style-based data augmentation, feature alignment or representation learning, frequency-domain generalization, and foundation-model-based approaches. Recently, Li et al. [13] proposed multivariate Fourier distribution perturbation to model domain shifts with uncertainty in frequency space. Similar to this line of work, SDU also explores stochastic spectral perturbation. However, SDU investigates this problem from a different perspective by interpreting medical domain shifts as uncertainty in spectral feature distributions. Unlike general Fourier augmentation, SDU is specifically designed for cross-domain medical image segmentation and performs uncertainty modeling on intermediate Transformer representations while preserving anatomical semantics through phase-aware reconstruction. In summary, current methods provide important insights into how to enlarge source-domain diversity and suppress domain-specific bias, but preserving anatomical structures while modeling realistic domain shifts remains a challenging problem.

### 2.2. Style-Based Data Augmentation

A common strategy for medical DG is to simulate unseen domains by augmenting the source-domain appearance. Early studies mainly relied on geometric transformations, intensity perturbations, or style augmentation to increase training diversity [7,14]. Although these methods are easy to implement, their perturbations are usually heuristic and may alter both domain-specific appearance and task-relevant anatomical structures. RAM-DSIR [7] further introduced random amplitude mixing and domain-specific image restoration for generalizable medical image segmentation, showing that amplitude-related style variation is beneficial for cross-domain robustness. More recent studies have explored more explicit style expansion mechanisms. ICRN [15] learns invariant content representations with local style augmentation to reduce style bias, while HSD [16] uses hallucinated style samples and distillation to encourage style-invariant feature learning. MoreStyle [17] relaxes the low-frequency constraint in Fourier-based image reconstruction to generate diverse source-domain styles. These methods demonstrate the effectiveness of style diversification, but most of them perform perturbation or reconstruction at the image level, where uncontrolled appearance changes may still affect structural consistency.

### 2.3. Feature Alignment and Representation Learning

Another line of work seeks to learn domain-invariant representations by aligning features across domains. Adversarial learning has been widely used for this purpose, where domain discriminators guide feature extractors to suppress domain-specific information [4,18]. Dou et al. [4] applied adversarial learning to cross-modality biomedical segmentation, but adversarial optimization may suffer from instability and mode collapse, especially when medical datasets are limited. DoFE [19] introduced domain-oriented feature embedding for generalizable fundus image segmentation, improving robustness on unseen datasets by modeling domain-related feature distributions. Recent methods further strengthen representation learning through content-style disentanglement or dynamic modeling. For example, ICRN [15] emphasizes invariant content extraction, and DDG [20] introduces dynamic domain generalization by adapting model behavior to simulate domain variations. These representation-based methods reduce source-domain overfitting to some extent, but they do not explicitly characterize domain shifts from a frequency-statistical perspective within transformer features.

### 2.4. Frequency-Domain Generalization

Frequency-domain analysis provides a useful perspective for separating structural information from appearance variations. Previous Fourier-based studies have shown that amplitude and phase components carry complementary information for domain transfer and generalization [8,9,21,22]. In general, phase information is closely related to spatial layout and structural geometry, whereas amplitude statistics are more sensitive to global appearance, intensity distribution, and style variation. Based on this observation, several recent medical DG methods have adopted frequency-domain perturbation. FreeSDG [23] mixes frequency components for single-source medical image segmentation, indicating that frequency-level augmentation can improve robustness under unseen domain shifts. RASS [24] synthesizes random amplitude spectra to expand source-domain appearance diversity. UniFreqSDG [25] further introduces universal frequency-domain perturbation with hierarchical feature-level modeling for single-source DG. DDG [20] also incorporates global-local Fourier random transformation into a dynamic generalization framework. These methods are closely related to our work because they confirm the importance of frequency perturbation for medical DG. However, most existing frequency-based methods either operate mainly at the image level or are designed for single-source generalization. In contrast, our method performs amplitude-statistics perturbation on intermediate transformer features, aiming to exploit feature-level frequency distributions while maintaining structural consistency through phase preservation.

### 2.5. Transformer Architectures and Foundation Models

Transformer-based architectures have shown strong potential in medical image segmentation due to their ability to model long-range dependencies. Vision Transformers [26] and hybrid CNN-Transformer models such as TransUNet [27] provide effective global context modeling for dense prediction tasks. Frequency-aware transformers have also been explored in medical segmentation; for example, Liu et al. [28] introduced frequency-aware interaction for ultrasound image segmentation. However, these methods are not primarily designed for domain generalization, and their frequency components are usually used for feature enhancement rather than controlled domain perturbation. Recently, foundation models have provided a new route for generalizable medical segmentation. DAPSAM [29] adapts the Segment Anything Model with domain-adaptive prototype prompts to improve cross-domain segmentation performance. Although such methods benefit from large-scale pretraining and prompt learning, they often require additional foundation-model components and prompt design. In contrast to these approaches, our MFP module is a lightweight training-phase plug-in for transformer-based segmentation networks. It perturbs amplitude statistics in the feature frequency space to simulate domain shifts, while preserving phase-related structural information for anatomically consistent segmentation.

## 3. Method

### 3.1. Problem Formulation

Given *K* source domains ${\left\{D_{s}^{k}\right\}}_{k=1}^{K}$, where each domain $D_{s}^{k}={\left\{\left(x_{i}^{k},y_{i}^{k}\right)\right\}}_{i=1}^{N_{k}}$ follows the distribution $P^{k}\left(x,y\right)$, the objective of domain generalization (DG) is to learn a segmentation model $f_{\theta }$ that generalizes well to an unseen target domain $D_{t}$:

(1) $$\min_{\theta }E_{\left(x,y\right)∼P^{s}}\left[L(f_{\theta }\left(x\right),y)\right],$$

where E[·] denotes the expectation operator, $P_{s}$ is the joint distribution of the source domains $D_{s}$, $f_{\theta }\left(\cdot \right)$ denotes the segmentation network parameterized by θ, θ represents the trainable network parameters, and L(·) denotes the segmentation loss function (Dice loss in our implementation).

The fundamental challenge arises from the distribution discrepancy between source and target domains, i.e.,

(2) $$P^{k}\left(x,y\right)\neq P^{t}\left(x,y\right),\;\forall k\in \left[1,K\right],$$

where $P_{t}$ is the is the joint distribution of the target domain $D_{t}$. Since target-domain data are unavailable during training, existing DG methods typically approximate such discrepancies through deterministic image transformations or feature perturbations. In contrast, we revisit medical domain shifts from a spectral perspective and model them as uncertainty in latent spectral distributions.

### 3.2. Spectral Uncertainty Hypothesis

Most existing DG approaches implicitly assume that unseen domains can be approximated by deterministic transformations of observed source-domain data. However, this assumption may be restrictive for medical imaging scenarios, where domain shifts arise from the complex interplay of scanner vendors, acquisition protocols, reconstruction pipelines, and patient populations.

We observe that although anatomical structures remain largely preserved across domains, substantial variations emerge in image appearance, texture, contrast, and noise characteristics. Such variations are predominantly reflected in spectral representations rather than structural content. This observation motivates the following hypothesis: Medical domain shifts are fundamentally characterized by uncertainty in spectral feature distributions. Consequently, unseen domains can be interpreted as stochastic realizations sampled from latent spectral distributions. Under this perspective, robust domain generalization can be achieved by exposing the model to diverse spectral realizations generated from uncertain spectral distributions during training. Based on this hypothesis, we propose Spectral-Distribution Uncertainty (SDU), a frequency-aware framework that explicitly models uncertainty in the Fourier amplitude distributions of intermediate representations.

### 3.3. Spectral Decomposition of Transformer Features

Let the input patches $x\in R^{N\times D}$ be the intermediate features from a vision Transformer [26] encoder block for each sample, where *N* denotes the sequence length (number of patches per image), and *D* is the embedding dimension. We apply a 1D Fourier Transform along the sequence dimension:

(3) $$F\left(x\right)\left(u,d\right)=\sum_{n=0}^{N-1}x\left(n\right)e^{-j2\pi un/N}=A_{x}\left(u,d\right)e^{jP_{x}\left(u,d\right)},$$

where $A_{x}\in R_{\geq 0}^{N\times D}$ represents the amplitude spectrum and $P_{x}\in {\left[-\pi ,\pi \right]}^{N\times D}$ denotes the phase spectrum of input image *x*. u∈{0,1,…,N−1} denotes the frequency index, and d∈{0,1,…,D−1} represents the embedding dimension index.

Previous studies have shown that the phase spectrum predominantly preserves spatial structures and semantic information, such as anatomical shapes, organ boundaries, and spatial layouts, whereas the amplitude spectrum is more sensitive to appearance-related variations, including contrast, texture, illumination, noise patterns, and scanner-specific characteristics [22,30]. Based on this observation, we perform uncertainty modeling in the amplitude space while preserving the original phase information.

### 3.4. Spectral-Distribution Uncertainty Modeling

Following the proposed hypothesis, we model spectral amplitude distributions as uncertain variables and simulate potential unseen domains through stochastic spectral sampling. For each frequency component *u*, we first compute the amplitude statistics (mean and standard deviation) of each image *x* along the embedding dimension:

(4) $$\mu_{x}\left(u\right)=\frac{1}{D}\sum_{d=1}^{D}A_{x}\left(u,d\right),$$

(5) $$\sigma_{x}\left(u\right)=\sqrt{\frac{1}{D}\sum_{d=1}^{D}{\left(A_{x}\left(u,d\right)-\mu_{x}\left(u\right)\right)}^{2}+\epsilon }.$$

where ϵ is a small positive constant introduced for numerical stability to avoid division by zero or extremely small denominators during variance estimation. $\mu_{x}\left(u\right)$ and $\sigma_{x}\left(u\right)$ are the *u*-th component of $\mu_{x}$ and $\sigma_{x}$, respectively, where $\mu_{x},\sigma_{x}\in R^{N\times 1}$ denote the frequency-wise mean and standard deviation of the amplitude spectrum for input image *x*, respectively. $\mu_{x},\sigma_{x}$ also represent the intra-sample spectral statistics, usually capturing the intrinsic spectral characteristics of each individual sample.

Subsequently, we use the intra-sample spectral statistics (i.e., $\mu_{x},\sigma_{x}$) within the batch to estimate the spectral uncertainty for each sample. We first calculate the inter-sample variance of $\mu_{x}$ and $\sigma_{x}$ within the input batch B. The process is formulated as:

(6) $$D_{\mu }\left(u\right)=E_{x\in B}\left[{\left(\mu_{x}\left(u\right)-E_{x\in B}\left[\mu_{x}\left(u\right)\right]\right)}^{2}\right],$$

(7) $$D_{\sigma }\left(u\right)=E_{x\in B}\left[{\left(\sigma_{x}\left(u\right)-E_{x\in B}\left[\sigma_{x}\left(u\right)\right]\right)}^{2}\right],$$

where B denotes the current mini-batch of training samples. Equations (6) and (7) compute the inter-sample variance of the frequency-wise mean $\mu_{x}$ and standard deviation $\sigma_{x}$ across all samples within the mini-batch, respectively. Specifically, $D_{\mu }$ measures the variability of the spectral mean among different samples, while $D_{\sigma }$ quantifies the variability of the spectral standard deviation. These two quantities characterize the uncertainty of spectral statistics across samples and are subsequently used to parameterize the Gaussian distributions for stochastic sampling.

Intuitively, larger variations indicate stronger uncertainty in spectral distributions and therefore larger potential domain shifts.

Then, the uncertainty of spectral statistics is modeled by Gaussian distributions over the estimated mean and standard deviation:

(8) $${\hat{\mu }}_{x}∼N\left(\mu_{x},D_{\mu }\right),{\hat{\sigma }}_{x}∼N\left(\sigma_{x},D_{\sigma }\right)$$

And ${\hat{\mu }}_{x},{\hat{\sigma }}_{x}$ are randomly sampled with reparameterization as follows:

(9) $${\hat{\mu }}_{x}\left(u\right)=\mu_{x}\left(u\right)+\lambda_{\mu }\sqrt{D_{\mu }\left(u\right)},\;\lambda_{\mu }∼N\left(0,1\right),$$

(10) $${\hat{\sigma }}_{x}\left(u\right)=\sigma_{x}\left(u\right)+\lambda_{\sigma }\sqrt{D_{\sigma }\left(u\right)},\;\lambda_{\sigma }∼N\left(0,1\right).$$

where $\lambda_{\mu }$ and $\lambda_{\sigma }$ are two random variables sampled from a standard Gaussian distribution, enabling differentiable stochastic sampling via the reparameterization trick. This formulation allows the model to efficiently generate various spectral statistics while maintaining end-to-end optimization.

The amplitude spectrum is reconstructed as:

(11) $${\hat{A}}_{x}\left(u,d\right)={\hat{\sigma }}_{x}\left(u\right)\frac{A_{x}\left(u,d\right)-\mu_{x}\left(u\right)}{\sigma_{x}\left(u\right)+\epsilon }+{\hat{\mu }}_{x}\left(u\right),$$

where the same ϵ is also used in Equation (5) to prevent being divided by zero when normalizing the amplitude spectrum. In all experiments, ϵ is empirically set to $10^{-6}$. Through stochastic sampling from uncertain spectral distributions, SDU synthesizes diverse yet plausible spectral realizations that emulate potential unseen domains.

### 3.5. Semantic-Preserving Reconstruction

After spectral perturbation, the modified amplitude spectrum is recombined with the original phase representation and transformed back into the feature space using the inverse Fourier transform:

(12) $$\hat{x}=F^{-1}\left({\hat{A}}_{x}e^{jP_{x}}\right).$$

Since the phase component remains unchanged, the reconstructed representation preserves anatomical structures and semantic content while exhibiting diverse spectral characteristics. Consequently, the network is encouraged to learn representations that are robust to uncertainty-induced spectral shifts, thereby improving generalization to unseen clinical domains.

The proposed SDU module is flexibly inserted after the multi-head self-attention layers in the shallow (i.e., the first three) encoder layers, providing a flexible and generalizable feature enhancement mechanism in the spectral domain. Layer normalization is applied both before and after SDU to stabilize training. During training, the gradient flow to phase components is stopped to preserve the structural integrity of the anatomical features. During the testing stage, SDU is discarded because it is merely required in the training stage, resulting in no additional inference computational overhead. For more detailed comprehension, the complete algorithm is provided in Algorithm 1.
**Algorithm 1:** The proposed Spectral-Distribution Uncertainty (SDU) modeling method**Require:** Intermediate Transformer encoder features $x\in R^{N\times D}$
**Ensure:** Generalized features $\hat{X}$
1:**Spectral Decomposition:**2:Compute FFT along sequence dimension via Equation (3)3:Decompose amplitude $A_{x}$ and phase $P_{x}$ using Equation (3)4:**Uncertainty Modeling:**5:**for** each frequency component *u*
**do**6:  Compute intra-sample statistics $\mu_{x}$ and $\sigma_{x}$ using Equations (4) and (5)7:  Estimate inter-sample variance $D_{\mu },D_{\sigma }$ via Equations (6) and (7)8:  Randomly sampling from Gaussian distribution via Equations (9) and (10)9:  Reconstruct perturbed amplitude spectrum using Equation and (11)10:**end for**11:**Semantic-Preserving Reconstruction:**12:Maintain original phase $P_{x}$ from Equation (3)13:Apply inverse FFT via Equation (12)


### 3.6. Theoretical Analysis

To analyze the behavior of the proposed stochastic perturbation, we consider the KL divergence between the perturbed-source spectral distribution and the original source spectral distribution. Let $P^{s}\left(A\right)$ and $P^{s}\left(\hat{A}\right)$ denote the amplitude distributions of the source and perturbed-source domains, respectively. Assuming that the spectral-distribution transformation is Lipschitz continuous with respect to spectral perturbations, the induced distribution discrepancy can be bounded as:

(13) $$\mathrm{KL}\left(P^{s}\left(A\right)∥P^{s}\left(\hat{A}\right)\right)\leq L^{2}E[∥\lambda_{\mu }\sqrt{D_{\mu }}+\lambda_{\sigma }\sqrt{D_{\sigma }}{∥}_{2}^{2}],$$

where $\left|\right|\cdot {\left|\right|}_{2}$ denotes the Euclidean norm, and KL(·∥·) denotes the Kullback–Leibler (KL) divergence, which measures the discrepancy between two probability distributions $P^{s}\left(A\right)$ and $P^{s}\left(\hat{A}\right)$. Specifically, the KL divergence is defined as: $\mathrm{KL}\left(P\left(z\right)∥Q\left(z\right)\right)=\int P\left(z\right)\log\frac{P(z)}{Q(z)}dz$. Here, the Lipschitz assumption characterizes the bounded sensitivity of the spectral-distribution transformation with respect to uncertainty perturbations. This analysis assumes that the spectral distributions are sufficiently smooth and that the perturbed distributions remain within the support of the original spectral feature space. Under these assumptions, Equation (13) provides an upper bound on the distribution discrepancy introduced by stochastic spectral perturbation. This analysis indicates that SDU adaptively controls the magnitude of spectral perturbations according to the estimated uncertainty. When the spectral statistics exhibit larger variations, SDU allows larger perturbations, encouraging the model to learn representations robust to potential spectral shifts.

Note that this analysis does not constitute a theoretical guarantee of target-domain generalization, since the target distribution is unavailable during training. Instead, it provides an interpretation of how uncertainty-guided perturbation expands the spectral feature distribution and controls the magnitude of generated spectral variations.

## 4. Experiments

### 4.1. Experimental Settings

#### 4.1.1. Datasets and Protocols

We evaluate the Spectral-Distribution Uncertainty modeling (SDU) method on three clinically significant datasets, each covering distinct medical modalities and anatomical structures, to ensure robustness across different medical imaging domains. The datasets used in our experiments are as follows:

- **Fundus** [19]: This dataset contains four retinal fundus domains annotated with optic cup/disc segmentations. The domain shift arises from variations in imaging devices (Zeiss vs. Topcon) and ethnic populations (Asian vs. European).
- **Prostate MRI** [31]: A six-domain dataset of T2-weighted MRI slices, which vary in slice thickness (3–5 mm) and magnetic field strengths (1.5T vs. 3T), leading to domain shifts.

Following the leave-one-domain-out protocol, we select one domain as the unseen target in each run, while the remaining domains serve as the source domains for training. All experiments are averaged over three runs to ensure reliable results.

#### 4.1.2. Implementation Details

Our SDU is integrated into different transformer backbones such as TransUNet [27] and SAM-UNet [32], leveraging its capacity to model global dependencies in medical image segmentation. Key experimental details are as follows:

- **Pre-processing**: For the Fundus dataset, images are center-cropped to 800 × 800 pixels and resized to 256 × 256. Prostate MRI slices are resampled to a resolution of 384 × 384.
- **Training**: We use the SGD optimizer with a learning rate of 0.01 and momentum of 0.9. The batch sizes are adapted per dataset: Fundus (8), Prostate (2), and BraTS (16). Early stopping is applied based on the validation loss.
- **Evaluation Metrics**: We use two standard metrics to evaluate segmentation performance: the Dice Similarity Coefficient (DSC) and the Average Surface Distance (ASD), both computed on the hold-out test sets. The DSC measures the spatial overlap between the predicted segmentation $S_{p}$ and the ground-truth segmentation $S_{g}$, which is defined as:
  (14) $$DSC=\frac{2|S_{p}\cap S_{g}|}{\left|S_{p}\right|+\left|S_{g}\right|},$$
  where |·| denotes the number of pixels/voxels in a segmentation region. A higher DSC indicates better segmentation overlap. The ASD measures the average distance between the boundaries of the predicted and ground-truth regions, defined as:
  (15) $$ASD=\frac{1}{\left|S_{p}\right|+\left|S_{g}\right|}\left(\sum_{p\in S_{p}}d\left(p,S_{g}\right)+\sum_{g\in S_{g}}d\left(g,S_{p}\right)\right),$$
  where d(·,·) denotes the shortest Euclidean distance between a boundary point and the opposite segmentation surface. A lower ASD indicates better boundary agreement.
- **Baselines**: For a fair comparison, we establish baseline models by training transformer-based backbones (TransUNet and SAM-UNet) using the conventional training strategy. Furthermore, we perform an additional baseline experiment by fine-tuning the baseline models with low-rank adaptation (LoRA) to evaluate the impact of parameter-efficient fine-tuning.

### 4.2. Comparison with State-of-the-Art Methods

We compare SDU against several leading Domain Generalization (DG) methods: DoFE [19], RAM-DSIR [7], DCAC [33], and DDG [20]. Additionally, we separately use two different networks (i.e., TransUNet and SAM-UNet) as the backbones to implement our method.

#### 4.2.1. Fundus Results (Table 1)

**Figure 2:**
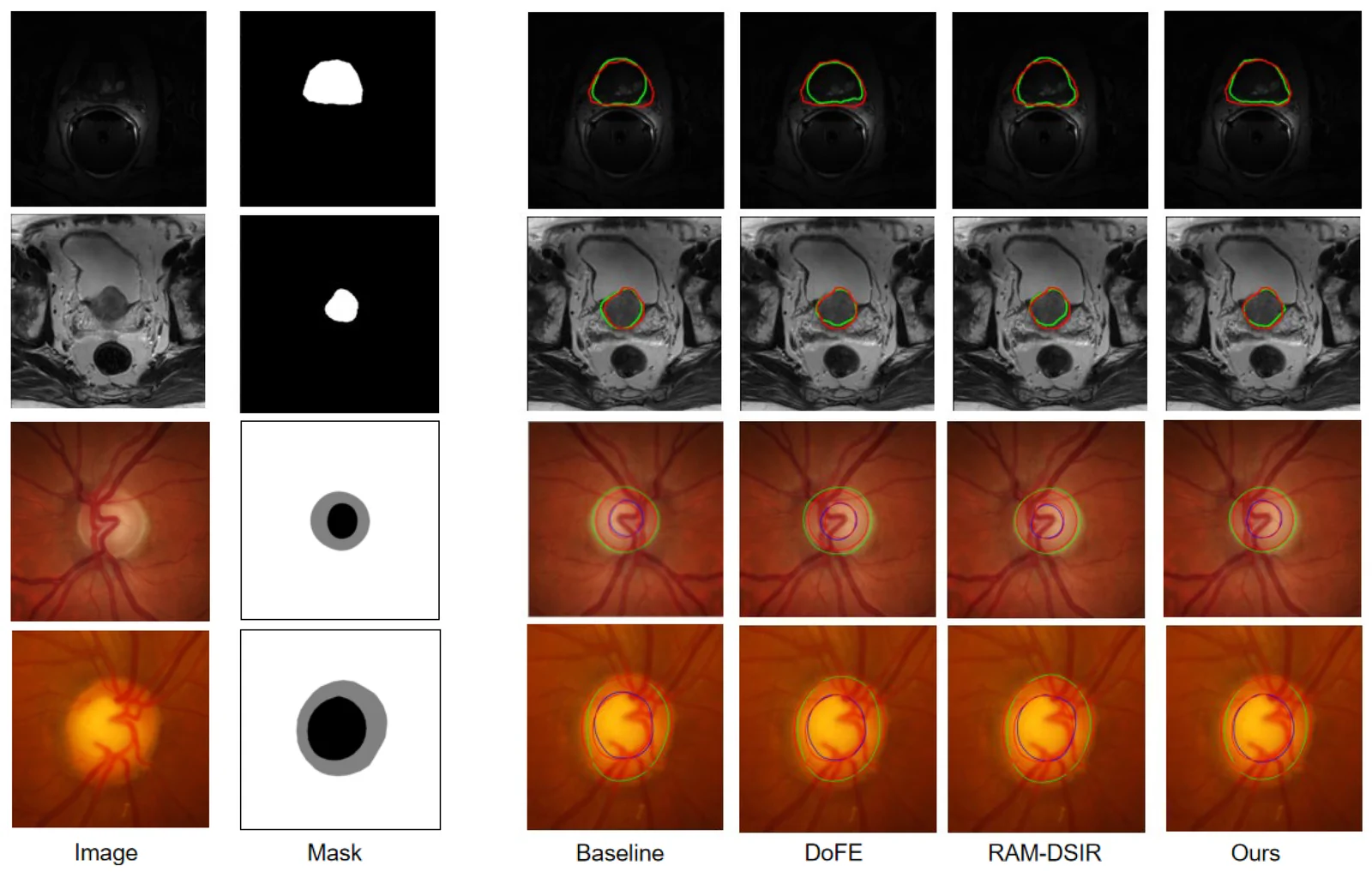
Visualization on segmentation results of different methods on **Prostate** datasets (**top two rows**) and **Fundus** (**bottom two rows**) dataset. The red contours indicate the boundaries of ground-truths, while the blue and green contours are the predictions of the model. The first column shows the original images, and the second column represents the mask with label information.

The results on the Fundus dataset demonstrate the effectiveness of the proposed SDU in improving domain generalization for optic cup/disc segmentation. Among all compared methods, our “LoRA+SDU” achieves the best overall performance, obtaining an average DSC of 88.68% and an average ASD of 10.66, outperforming existing domain generalization methods, including DoFE, RAM-DSIR, DCAC, and DDG. Furthermore, SDU consistently improves segmentation performance under different backbone architectures. Compared with LoRA fine-tuning alone, LoRA+SDU further improves DSC from 88.03% to 88.68% and reduces ASD from 11.34 to 10.66, demonstrating that SDU provides complementary benefits beyond parameter-efficient fine-tuning. This performance improvement is attributed to the amplitude perturbations in SDU that enhance domain invariance of learned feature representations. The qualitative results in Figure 2 further verify that the proposed method maintains anatomical structures while improving segmentation robustness under unseen domains.

**Table 1:** Comparison of different methods on **Fundus** dataset segmentation task. The reported results follow the multi-source leave-one-domain-out protocol. The best results are **bolded**.

Backbone	Task	Optic Cup (OC)/Optic Disc (OD) Segmentation					
	Unseen Domain	Domain 1	Domain 2	Domain 3	Domain 4	OC/OD Avg.	Overall Avg.
	Method	Dice Coefficient (DSC) ↑					
UNet	DoFE [19]	80.07/94.90	75.52/89.08	84.72/92.28	87.26/93.85	81.89/92.53	87.21
	RAM-DSIR [7]	82.09/95.27	77.40/87.17	84.18/94.66	83.44/93.94	81.78/92.76	87.27
	DCAC [33]	81.43/96.54	77.72/87.85	86.80/94.28	87.68/95.40	83.41/93.52	88.47
	DDG [20]	84.27/95.19	76.46/87.54	83.99/93.72	85.20/94.25	82.48/92.68	87.57
TransUNet	Baseline	81.29/95.44	77.44/89.68	82.78/93.06	81.81/91.04	80.83/92.31	86.57
	Baseline + SDU (Ours)	82.51/95.89	79.84/91.30	86.94/94.29	81.14/90.44	82.61/92.98	87.79
SAM-Unet	Baseline	75.61/96.24	80.98/88.94	86.83/94.57	79.42/91.79	80.71/92.89	86.8
	Baseline + SDU (Ours)	78.58/96.54	78.47/89.43	85.20/95.08	82.34/90.71	81.15/92.94	87.05
	LoRA	83.53/96.14	78.94/89.32	86.35/95.32	82.76/91.84	82.90/93.16	88.03
	LoRA + SDU (Ours)	84.47/96.55	80.28/90.69	87.13/94.81	82.65/92.88	83.63/93.73	**88.68**
		**Average Surface Distance (ASD) ↓**					
UNet	DoFE [19]	21.98/8.85	18.13/16.16	10.73/10.78	6.76/6.81	14.40/10.65	12.53
	RAM-DSIR [7]	18.51/8.45	16.62/18.50	10.96/7.60	17.01/6.95	15.78/10.38	13.08
	DCAC [33]	19.20/6.35	17.15/18.28	9.14/8.11	7.12/5.20	13.15/9.49	11.32
	DDG [20]	15.85/8.58	17.37/18.99	11.09/9.09	11.54/7.26	13.96/10.98	12.47
TransUNet	Baseline	20.06/7.92	16.90/16.34	11.71/10.74	10.77/10.08	14.86/11.27	13.06
	Baseline + SDU (Ours)	17.73/7.26	14.16/13.33	9.30/8.12	11.11/10.37	13.08/9.77	11.42
SAM-Unet	Baseline	25.40/6.72	15.15/16.39	9.68/7.75	12.63/10.69	15.72/10.39	13.05
	Baseline + SDU (Ours)	22.01/6.15	15.50/15.77	10.38/7.02	10.16/10.68	14.51/9.91	12.21
	LoRA	15.34/6.71	15.33/17.08	9.79/7.24	9.57/9.63	12.51/10.17	11.34
	LoRA + SDU (Ours)	15.05/6.25	12.99/15.36	9.31/7.13	10.54/8.61	11.97/9.34	**10.66**

#### 4.2.2. Prostate MRI Results (Table 2)

Among all compared methods, our LoRA+SDU achieves the best overall performance, obtaining an average DSC score of 89.03% and the lowest average ASD of **1.22**. Compared with existing domain generalization methods, LoRA+SDU improves the average DSC by 1.16% and 1.22% over DCAC (88.61%) and RAM-DSIR (87.81%), respectively, while reducing ASD by 0.19 and 0.26 compared with DCAC and RAM-DSIR. To investigate the contribution of SDU under different backbone architectures, we further compare multiple training strategies based on TransUNet and SAM-Unet. For TransUNet, incorporating SDU improves the average DSC from 87.22% to 88.05% and reduces ASD from 1.65 to 1.38. Similarly, when applied to SAM-Unet, SDU consistently improves performance, increasing the average DSC from 87.67% to 88.34% and reducing ASD from 1.89 to 1.67. These results demonstrate that SDU provides stable improvements regardless of the underlying segmentation architecture, highlighting its effectiveness as a general domain enhancement strategy. Furthermore, combining SDU with LoRA achieves additional performance gains. Compared with LoRA fine-tuning alone, LoRA+SDU improves the average DSC from 88.26% to **89.03%** and reduces ASD from 1.54 to 1.22. These improvements indicate that the proposed method can effectively learn domain-invariant representations and achieve more accurate prostate boundary delineation under unseen domains.

**Table 2:** Comparison of different methods on **Prostate** dataset segmentation task. The reported results follow the multi-source leave-one-domain-out protocol. The best results are **bolded**.

Backbone	Task	Prostate Segmentation						
	Unseen Domain	Domain 1	Domain 2	Domain 3	Domain 4	Domain 5	Domain 6	Avg.
		Dice Coefficient (DSC) ↑						
UNet	DoFE [19]	88.12	88.23	85.13	89.15	85.12	86.98	87.12
	RAM-DSIR [7]	87.48	90.08	**86.34**	88.09	86.98	87.86	87.81
	DCAC [33]	**91.76**	90.51	86.30	89.13	83.39	**90.56**	88.61
	DDG [20]	84.71	90.13	84.15	87.69	87.48	87.24	86.90
TransUNet	Baseline	89.94	90.72	80.91	88.01	85.19	88.54	87.22
	Baseline + SDU (Ours)	90.78	91.12	85.14	88.18	83.25	89.82	88.05
SAM-Unet	Baseline	90.27	90.36	81.43	89.04	86.04	88.87	87.67
	Baseline + SDU (Ours)	90.44	90.84	83.42	**89.39**	87.06	88.90	88.34
	LoRA	89.75	90.81	83.77	89.20	87.38	88.63	88.26
	LoRA + SDU (Ours)	90.59	**91.41**	85.45	88.97	**88.43**	89.32	**89.03**
		**Average Surface Distance (ASD) ↓**						
UNet	DoFE [19]	0.87	1.30	2.89	1.54	2.31	1.24	1.69
	RAM-DSIR [7]	1.09	0.91	**2.38**	1.25	1.95	1.27	1.48
	DCAC [33]	0.98	0.89	1.77	1.53	2.46	0.85	1.41
	DDG [20]	1.22	0.76	3.10	1.36	1.92	0.83	1.53
TransUNet	Baseline	0.84	0.76	4.52	1.34	1.72	0.72	1.65
	Baseline + SDU (Ours)	**0.72**	0.63	2.74	1.68	1.85	**0.63**	1.38
SAM-Unet	Baseline	0.79	0.70	6.46	0.97	1.76	0.68	1.89
	Baseline + SDU (Ours)	0.78	0.66	5.24	1.13	1.55	0.67	1.67
	LoRA	0.75	0.78	4.33	1.01	1.68	0.70	1.54
	LoRA + SDU (Ours)	0.77	**0.61**	3.07	**0.81**	**1.43**	0.65	**1.22**

### 4.3. Ablation Studies

#### 4.3.1. Module Insertion Strategy (Table 3 and Table 4)

An ablation study systematically evaluates the contribution of individual components by modifying or removing one component at a time while keeping all other settings unchanged. In this work, the ablation study is performed to investigate the effect of different SDU insertion positions (Table 3 and Table 4). Our ablation studies demonstrate that applying SDU to the first three encoder layers (1–3) into TransUNet achieves the best overall performance, obtaining the highest DSC score and the lowest ASD on both Fundus and Prostate datasets. In contrast, inserting SDU into deeper layers (e.g., 1–12) results in a slight performance degradation. This observation is consistent with the information bottleneck principle [34], where shallow layers generally retain low-level appearance statistics that are closely associated with domain-specific variations, whereas deeper layers progressively encode more general and task-relevant semantic representations. Since SDU performs feature distribution perturbation by modeling and sampling from estimated feature statistics, applying it to shallow layers enables effective simulation of domain-specific variations while preserving semantic information. Conversely, perturbing deeper representations may introduce undesirable alterations to task-discriminative features, leading to weakened structural consistency and a slight decline in segmentation performance compared with the optimal performance.

**Table 3:** Ablation study on different insertion positions of the SDU module in TransUNet for the **Fundus** dataset. Each entry reports the DSC (%) of optic cup (OC)/optic disc (OD) segmentation under four unseen-domain evaluation settings, and the OC/OD Avg. column denotes the average DSC over all unseen domains in terms of optic cup and optic disc segmentation. SDU (1–k) denotes inserting the SDU module after the k-th ViT encoder block.

Unseen Domain	Domain 1	Domain 2	Domain 3	Domain 4	OC/OD Avg.	Overall Avg.
TransUNet	81.29/95.44	77.44/89.68	82.78/93.06	81.81/91.04	80.83/92.31	86.57
+ SDU (1–1)	83.20/95.15	79.39/90.59	84.56/92.92	81.33/91.88	82.12/92.64	87.38
+ SDU (1–2)	83.89/95.08	80.41/90.42	84.47/92.16	84.03/90.84	83.2/92.12	87.66
+ SDU (1–3)	82.51/95.89	79.84/91.30	86.94/94.29	81.14/90.44	82.61/92.98	**87.79**
+ SDU (1–4)	84.69/96.61	80.05/90.75	86.53/92.12	81.27/88.96	83.13/92.11	87.62
+ SDU (1–5)	83.72/95.83	80.21/90.95	85.04/91.70	83.05/88.75	83.00/91.81	87.41
+ SDU (1–6)	86.30/95.27	79.05/90.59	81.46/92.76	81.53/89.15	82.08/91.94	87.01
+ SDU (1–7)	84.26/94.98	79.68/88.91	84.49/91.39	82.51/91.77	82.73/91.76	87.25
+ SDU (1–8)	82.77/95.89	81.12/90.05	84.66/91.64	83.32/91.02	82.97/92.15	87.55
+ SDU (1–9)	81.82/95.32	80.62/90.08	84.08/94.63	84.09/89.09	82.65/92.28	87.47
+ SDU (1–10)	84.73/96.06	80.39/90.25	82.26/93.16	82.05/91.13	82.36/92.65	87.51
+ SDU (1–11)	85.95/96.07	79.56/90.36	83.87/93.71	80.81/88.11	82.55/92.06	87.31
+ SDU (1–12)	82.79/95.88	80.22/90.59	84.69/93.81	80.14/92.08	81.96/93.09	87.53

**Table 4:** Ablation study on different insertion positions of the SDU module in TransUNet for the **Prostate** dataset. Each entry reports the DSC (%) prostate segmentation under six unseen-domain evaluation settings, and the Avg. column denotes the average DSC over all unseen domains.

Unseen Domain	Domain 1	Domain 2	Domain 3	Domain 4	Domain 5	Domain 6	Avg.
TransUNet	89.94	90.72	80.91	88.01	85.19	88.54	87.22
+ SDU (1–1)	90.11	90.98	81.43	88.39	85.87	88.32	87.52
+ SDU (1–2)	90.68	91.19	81.49	89.17	85.44	88.93	87.82
+ SDU (1–3)	90.78	91.12	85.14	88.18	83.25	89.82	**88.05**
+ SDU (1–4)	91.16	91.11	84.12	88.20	84.38	88.97	87.99
+ SDU (1–5)	90.56	90.76	83.54	88.78	84.56	89.35	87.93
+ SDU (1–6)	90.37	90.88	83.30	88.78	85.09	89.19	87.94
+ SDU (1–7)	90.39	90.62	83.19	88.55	86.46	89.04	88.04
+ SDU (1–8)	90.15	90.47	83.08	88.58	87.58	88.20	88.01
+ SDU (1–9)	90.08	90.65	83.49	88.02	87.22	88.54	88.00
+ SDU (1–10)	90.10	90.53	83.38	88.73	86.07	88.89	87.95
+ SDU (1–11)	90.08	90.29	83.77	88.13	86.19	88.48	87.82
+ SDU (1–12)	90.28	91.23	81.57	89.47	84.34	88.95	87.64

#### 4.3.2. Perturbation Intensity Analysis (Figure 3)

**Figure 3:**
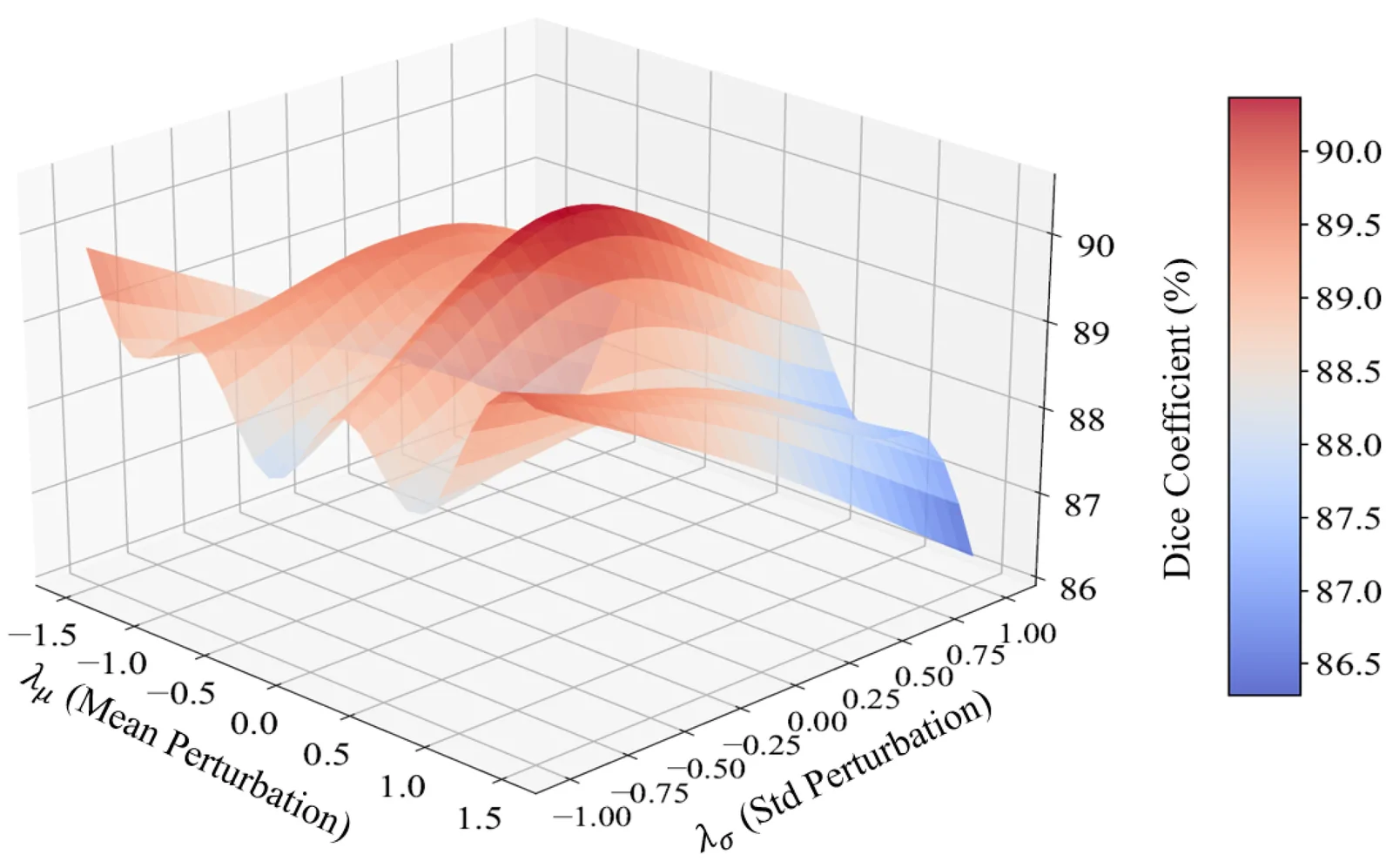
Impact of perturbation strength ($\lambda_{\mu },\lambda_{\sigma }$) on Fundus dataset.

We systematically analyze the effects of the perturbation coefficients $\lambda_{\mu }$ (mean shift) and $\lambda_{\sigma }$ (standard deviation scaling) as defined in Equations (9) and (10) on the Fundus dataset. The experiments are conducted on TransUNet equipped with our SDU module in the first three encoder layers. The results in Figure 3 reveal that sampling $\lambda_{\mu },\lambda_{\sigma }∼N\left(0,1\right)$ yields the best performance, achieving a DSC score of 88.05% and an Average Surface Distance (ASD) of 1.38 mm. Three distinct regimes are observed based on the values of $\lambda_{\mu }$ and $\lambda_{\sigma }$:

- In the high-fidelity zone ($\left|\lambda_{\mu }\right|<0.5$ and $\left|\lambda_{\sigma }\right|<0.3$), the model maintains a stable performance with an average DSC score of 87.2% (±0.8%);
- In the semantic distortion zone ($\lambda_{\mu }>1.0$), there is an exponential increase in ASD, with a 15.2% increase observed when $\lambda_{\mu }$ reaches 1.5;
- In the under-perturbation zone ($\lambda_{\mu }<-0.8$), the reduced perturbation leads to diminished cross-domain diversity, resulting in a DSC decrease of 2.3%.

In particular, the analysis reveals an asymmetric sensitivity, with the response to mean perturbation ($\lambda_{\mu }$) being 30% more pronounced than that to standard deviation scaling ($\lambda_{\sigma }$), demonstrating the dominant role of mean perturbation in improving feature representations.

### 4.4. Qualitative Analysis

#### 4.4.1. Feature Space Visualization (Figure 4)

**Figure 4:**
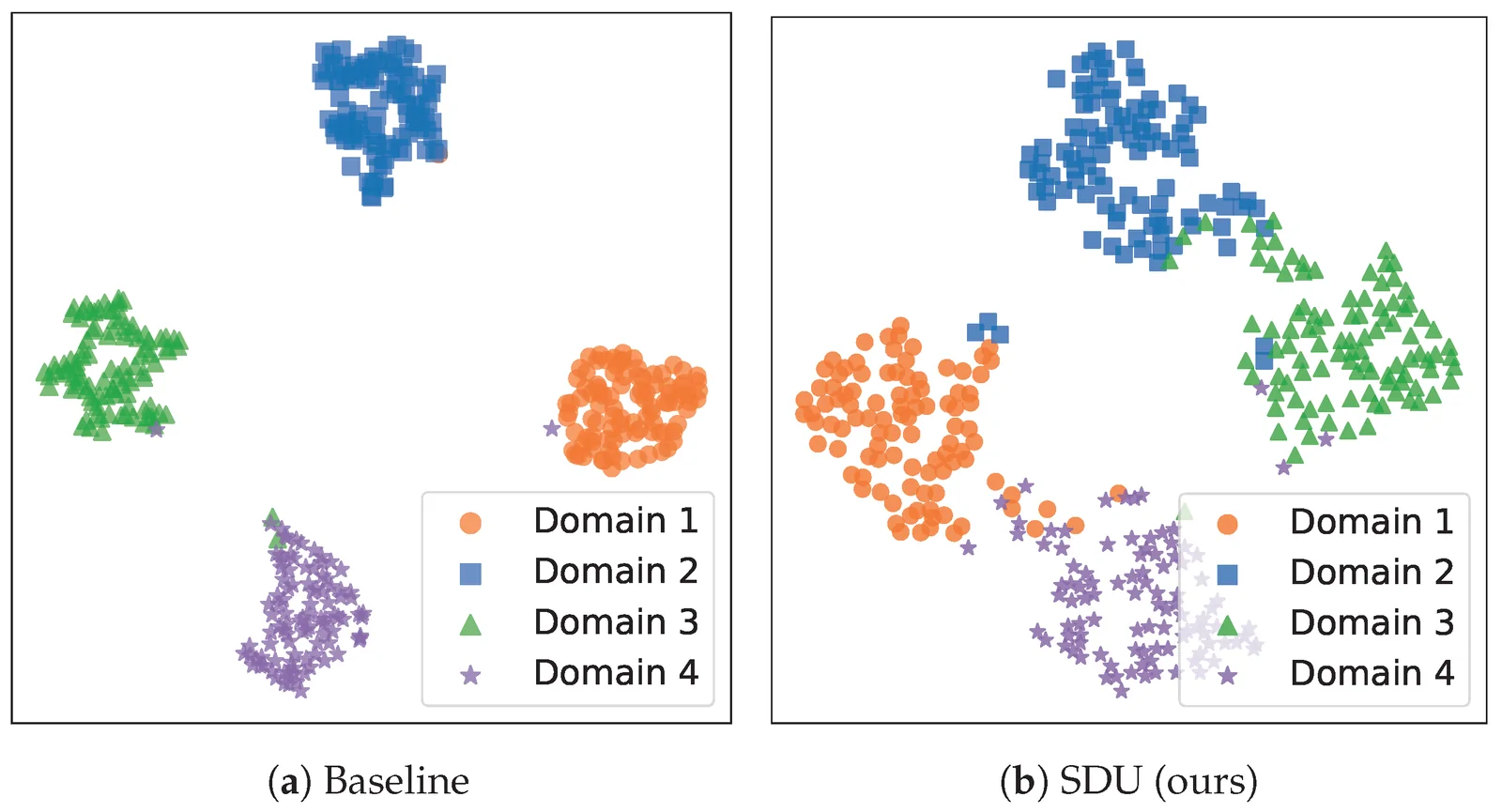
t-SNE visualization of features separately provided by the baseline and our SDU model fed with the images from the Fundus dataset, where Domain 1–3 are the training set and Domain 4 is the testing set.

We further investigate the effect of SDU on feature distribution alignment through t-SNE visualization. Specifically, features extracted by the baseline and SDU models are visualized on the Fundus dataset, where Domains 1–3 serve as source domains and Domain 4 is treated as the unseen target domain. As shown in Figure 4, the baseline model exhibits clear domain-specific clustering, with the target-domain features deviating from the source-domain distribution. In contrast, SDU generates a more continuous and diversified feature distribution, enabling the unseen Domain 4 samples to fall within the feature manifold spanned by the source domains. Since t-SNE is a nonlinear dimensionality-reduction technique, apparent overlaps in the two-dimensional embedding do not necessarily imply exact overlap in the original high-dimensional feature space. Therefore, the t-SNE visualization is used only as qualitative evidence of increased domain mixing. To quantitatively assess domain discrepancy, we additionally evaluate the inter-domain feature distance using Maximum Mean Discrepancy (MMD) [35]. Quantitatively, SDU reduces the inter-domain discrepancy by 38% in terms of MMD. These observations suggest that spectral uncertainty modeling allows SDU to estimate and perturb domain-specific feature statistics, effectively expanding the coverage of possible domain distributions while preserving semantic consistency. Consequently, the model obtains stronger robustness against unseen domain shifts.

#### 4.4.2. Computational Efficiency

SDU introduces only 0.8M additional parameters (1.2% of the total parameters in TransUNet) and adds a minimal computational overhead of 3ms per iteration, making it suitable for real-time deployment.

### 4.5. Discussion

Although the numerical improvements appear modest, domain generalization for medical image segmentation is inherently a highly saturated problem, where strong baselines already achieve high segmentation accuracy. Under such conditions, even small but consistent improvements across multiple unseen domains are generally considered meaningful. More importantly, SDU consistently improves segmentation performance across different datasets, backbone architectures, and unseen-domain evaluation settings, indicating that the proposed uncertainty modeling strategy provides stable generalization benefits rather than dataset-specific gains. From a clinical perspective, even small improvements in segmentation accuracy may translate into more reliable anatomical boundary delineation. For example, more accurate optic cup segmentation can improve the estimation of the cup-to-disc ratio, an important biomarker for glaucoma assessment, while more accurate prostate boundaries may benefit downstream tasks such as treatment planning and volumetric analysis. Furthermore, the proposed SDU module is lightweight and can be seamlessly integrated into different segmentation backbones without introducing substantial architectural modifications or computational overhead. Therefore, the observed performance gains are achieved with minimal additional complexity.

## 5. Conclusions

In this work, we propose a novel **Spectral-Distribution Uncertainty (SDU)** method for domain-generalizable medical image segmentation. In contrast to existing approaches that simulate domain shifts through predefined deterministic transformations, we revisit medical domain variations from a spectral perspective and hypothesize that unseen domain shifts can be characterized by uncertainty in spectral feature distributions. Based on this insight, SDU models the uncertainty of Fourier amplitude statistics in intermediate representations and introduces stochastic spectral perturbations to generate diverse yet anatomically consistent feature distributions. By preserving phase information while perturbing amplitude-related statistics, SDU improves domain robustness without compromising semantic structures.

Extensive experiments on retinal fundus and prostate MRI segmentation benchmarks demonstrate that SDU consistently enhances the generalization performance of different segmentation architectures. Quantitative comparisons, visualization analysis, and ablation studies further verify that SDU effectively reduces domain discrepancy and learns more robust representations under unseen domain shifts.

Overall, this study provides a new perspective that medical domain shifts are not merely deterministic appearance variations but can be modeled as uncertainty in spectral feature distributions. The proposed method offers a lightweight and effective solution for improving cross-domain generalization in medical image segmentation. Future work will explore more adaptive uncertainty modeling strategies and extend spectral-distribution learning to broader clinical scenarios and foundation-model-based segmentation systems.

## Data Availability

The Fundus benchmark for optic disc/cup segmentation aggregates three public retinal fundus datasets: Drishti-GS, RIM-ONE-r3, and REFUGE. REFUGE is split into two domains (training and validation subsets) due to different acquisition cameras. All images are available at https://zenodo.org/records/8009107?utm_source=chatgpt.com, accessed on 23 September 2026. The Prostate benchmark is the multi-site T2-weighted MRI dataset from the SAML work, which is available at https://liuquande.github.io/SAML/, accessed on 23 September 2026.

## Abbreviations

**Abbreviation**	**Type**	**Definition**
SDU	Proposed method	Spectral-Distribution Uncertainty
DoFE	Baseline method	Domain-aware Frequency Enhancement
RAM-DSIR	Baseline method	Random Amplitude Modulation with Domain Shift-Invariant Representation
DCAC	Baseline method	Domain Consistency Alignment Constraint
DDG	Baseline method	Dynamic Domain Generalization
ViT	Backbone	Vision Transformer
SAM	Backbone	Segment Anything Model
LoRA	Fine-tuning	Low-Rank Adaptation
DG	Learning paradigm	Domain Generalization
DSC	Evaluation metric	Dice Similarity Coefficient
ASD	Evaluation metric	Average Surface Distance
KL	Mathematical concept	Kullback–Leibler Divergence
MMD	Distance metric	Maximum Mean Discrepancy
